# Estimation of time course in phytochrome photostationary state under artificial light for controlling plant growth

**DOI:** 10.3389/fpls.2023.1305182

**Published:** 2024-01-24

**Authors:** Tomohiro Jishi

**Affiliations:** Grid Innovation Research Laboratory, Central Research Institute of Electric Power Industry, Chiba, Japan

**Keywords:** artificial lighting, dose response, end-of-day far-red light, low fluence response, model

## Abstract

A model to estimate the time course of a phytochrome photostationary state (PSS) under an arbitrary light environment was developed. It is the solution of differential equations that use conversion rates between active and inactive forms of previously reported phytochromes. The model estimated that 90% of the PSS changes were completed using approximately 3.4 mmol m^-2^ of integrated end-of-day far-red light irradiation, and 99% of the changes were completed with approximately 6.9 mmol m^-2^ irradiation. Although these values were affected by the spectral photon flux density of the far-red light. They were consistent with previous results that examined dose requirements of far-red irradiation. The rate at which the PSS changes approached equilibrium was maximized under a red light, followed by far-red, green, and blue light. This estimation method could be used to control phytochrome responses for horticulture via artificial lighting.

## 1 Introduction

Phytochromes are photoreceptors that have major effects on plant development and morphogenesis, including germination (Borthwick et al., 1952; Mancinelli et al., 1966), bud formation for flowers (Halliday et al., 1994), and stem elongation (Smith and Whitelam, 1997). In particular, phytochrome B changes reversibly between inactive (P_r_) and active (P_fr_) states. The P_r_ state has an absorption maximum at red wavelengths, which change it to P_fr,_. Whereas, P_fr_ has an absorption maximum at far-red wavelengths, which converts it to P_r_. The ratio of P_r_ to P_fr_ varies with the relative spectral photon flux density distribution (RSPFD), thereby serving as a sensor for the RSPFD. Promoted stem elongation, suppressed leaf development, and promoted flower bud formation under a high proportion of far-red (FR) light are characteristics of a shade-avoidance response (Folta and Carvalho, 2015).

There have been attempts to control plant growth, development, and morphology by regulating phytochrome B (hereinafter, simply referred to as phytochrome) reaction via artificial lighting. Blom et al. (1995) induced stem elongation in greenhouse-grown lilies via end-of-day far-red light (EODFR). Mata and Botto (2009) reported that poinsettia flowering was delayed by using a high percentage of red light produced with a film that absorbed far-red light. The widespread use of light-emitting diodes (LEDs) has facilitated narrow-band lighting, resulting in more reports on plant responses mediated by phytochromes (e.g., Chia and Kubota, 2010).

In these reports, the R/FR ratio of red light to far-red light in the photon flux density (PFD) and the phytochrome photostationary state (PSS) have been used as indicators of environmental light effects on the phytochrome status. Red and far-red light have been defined as having wavelengths over 600–700 nm and 700–800 nm, respectively (Yang et al., 2012; Shibuya et al., 2023), but sometimes the wavelengths were defined as 655–665 nm and 725–735 nm (Smith, 1986), or 660–670 nm and 725–735 nm (Franklin, 2008).

The PSS, sometimes called the phytochrome photo-equilibrium, is the ratio of active phytochrome to the total phytochrome (P_fr_/P_all_), and can be calculated from absorptivity data of isolated phytochromes (Sager et al., 1988). P_r_ and P_fr_ can also change their state by absorbing blue and green light. Some reports have suggested that monochromatic blue light affects plant morphology by reducing the PSS (Hernández and Kubota, 2016; Jishi et al., 2021a; Jishi et al., 2021b). When using blue or green light, the R/FR ratio is not a suitable indicator of the phytochrome reaction and PSS should be used instead.

When the light environment changes over a short timescale, phenomena occur that cannot be explained solely by calculating the steady-state PSS. Even though the calculated steady-state PSS is independent of the photon flux density (PFD), and is determined solely by the RSPFD, higher doses (=integrated PFD) of EODFR, produced longer hypocotyl lengths in tomato seedlings (Chia and Kubota, 2010). The hypocotyl elongation was saturated at 4 mmol m^-2^ s^-1^ EODFR doses, which could have been attributed to temporal changes in the PSS. Because PSS changes can take several minutes to complete (Quail, 1983), it has been suggested that a higher PFD produces faster PSS changes *in vivo* (Spruit and Kendrick, 1972). Therefore, if the dose, which is the product of PFD and the irradiation time, was not sufficient for the PSS to reach a steady state, the PSS change could stop midway.

If the PSS temporal changes could be estimated, then the effects of artificial lighting on plants via the action of the phytochrome could be estimated in more detail, and plant morphology and development could be controlled more efficiently and accurately. For example, there have been few effects on the PSS if the irradiation was continued after the steady state was reached, and energy consumption could be reduced by providing sufficient irradiation as needed (Chia and Kubota, 2010; Zou et al., 2021). In addition, one could attempt to stop the PSS change midway by adjusting the light irradiation time.

Here, a method for estimating temporal changes in the PSS is discussed. By using previously reported spectral data for phytochrome photochemical cross-sections, reaction rate constants for each change between P_r_ and P_fr_ were calculated, and the differential equations were solved. In addition, examples of model estimation results are discussed and compared with previously reported measurements.

## 2 Calculation method

### 2.1 Definition of phytochrome photochemical cross-section and PSS calculation

The phytochrome photochemical cross-section was defined as the conversion rate constant (m^2^ mol^-1^) for the PFD at each wavelength. Therefore, the rate of decrease in the PSS per unit time because of the inactivation conversion of P_fr_ to P_r_ is defined in Equation 1, and the rate of increase in the PSS per unit time because of the activation conversion of P_r_ to P_fr_ is defined in Equation 2:


(1) $$\begin{array}{c} \frac{dP}{dt}=-P\times \sum_{\lambda =300}^{800}E_{\text{λ}}{\text{σ}}_{\text{frλ}} \end{array}$$

(2) $$\begin{array}{c} \frac{dP}{dt}=\left(1-P\right)\times \sum_{\lambda =300}^{800}E_{\text{λ}}{\text{σ}}_{\text{rλ}} \end{array}$$

In Equations 1, 2, *P* is PSS, λ is the wavelength (nm), *E*_λ_ is the spectral photon flux density at λ (mol m^-2^ s^-1^ nm^-1^), and σ_rλ_ and σ_frλ_ are the phytochrome photochemical cross-sections (m^2^ mol^-1^) of P_r_ and P_fr_, respectively, at λ.

After a sufficient time and a constant RSPFD, these reaction rates were balanced, and Equation 3 could be assumed:


(3) $$\begin{array}{c} P\times \sum_{\lambda =300}^{800}E_{\text{λ}}{\text{σ}}_{\text{frλ}}=\;\left(1-P\right)\times \sum_{\lambda =300}^{800}E_{\text{λ}}{\text{σ}}_{\text{rλ}} \end{array}$$

By solving Equation 3, the steady-state PSS could be formulated as Equation 4 (Sager et al., 1988):


(4) $$\begin{array}{c} P=\frac{\sum_{\lambda =300}^{800}E_{\text{λ}}{\text{σ}}_{\text{frλ}}}{\sum_{\lambda =300}^{800}E_{\text{λ}}{\text{σ}}_{\text{rλ}}+\sum_{\lambda =300}^{800}E_{\text{λ}}{\text{σ}}_{\text{frλ}}} \end{array}$$

### 2.2 Method for estimating temporal changes in the PSS

Based on the above, temporal changes in PSS could be expressed as:


(5) $$\begin{array}{c} \frac{dP}{dt}=\;-P\times \sum_{\lambda =300}^{800}E_{\text{λ}}{\text{σ}}_{\text{frλ}}\;+\;\left(1-P\right)\times \sum_{\lambda =300}^{800}E_{\text{λ}}{\text{σ}}_{\text{rλ}} \end{array}$$

To simplify Equation 5, the sums were replaced by *a* and *b*:


(6) $$\begin{array}{c} a=\sum_{\lambda =300}^{800}E_{\text{λ}}{\text{σ}}_{\text{frλ}} \end{array}$$

(7) $$\begin{array}{c} b=\sum_{\lambda =300}^{800}E_{\text{λ}}{\text{σ}}_{\text{rλ}} \end{array}$$

Then, by solving Equation 5, the following was obtained:


(8) $$\begin{array}{c} P=\frac{b}{a+b}-C\;e^{-\left(a+b\right)t} \end{array}$$

where *C* is the constant of integration and e is the base of the natural logarithm. Defining *P*_0_ as *P* at *t* = 0, Equation 9 was obtained:


(9) $$\begin{array}{c} C=\frac{b}{a+b}-P_{0} \end{array}$$

Therefore, Equation 8 could be expressed as:


(10) $$\begin{array}{c} P=\frac{b}{a+b}-\left(\frac{b}{a+b}-P_{0}\right)e^{-\left(a+b\right)t} \end{array}$$

After substituting data for the spectral light and the phytochrome photochemical cross-sections into Equations 6, 7, and substituting a, b, and the initial PSS into Equation 10, the temporal change in PSS could be estimated.

## 3 Calculation examples

### 3.1 End-of-day far-red light

**Figure 1:**
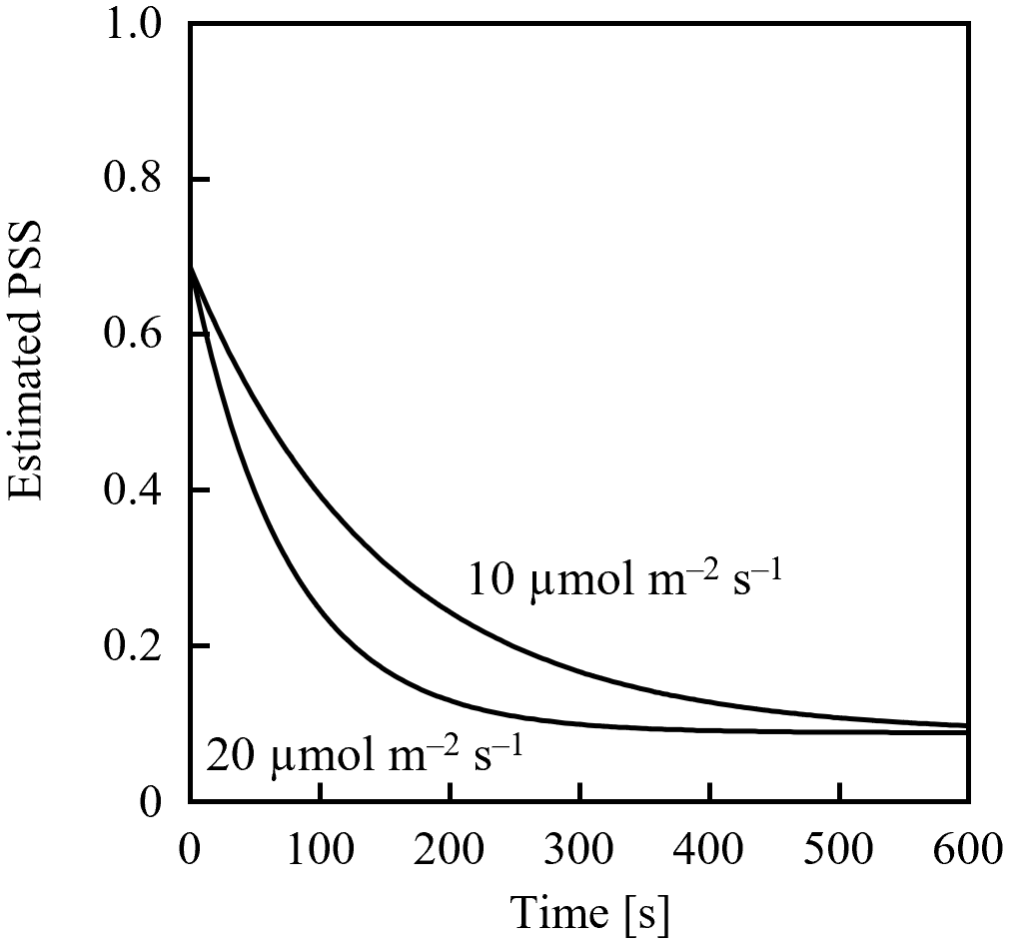
Estimated temporal changes in the phytochrome steady state (PSS) when 10- or 20-µmol m^-2^ s^-1^ end-of-day far-red lighting was applied. A spectral photon flux density distribution of a far-red LED with a 745-nm peak wavelength was used, as well as spectral data of the phytochrome photochemical cross-section (Sager et al., 1988).

**Figure 2:**
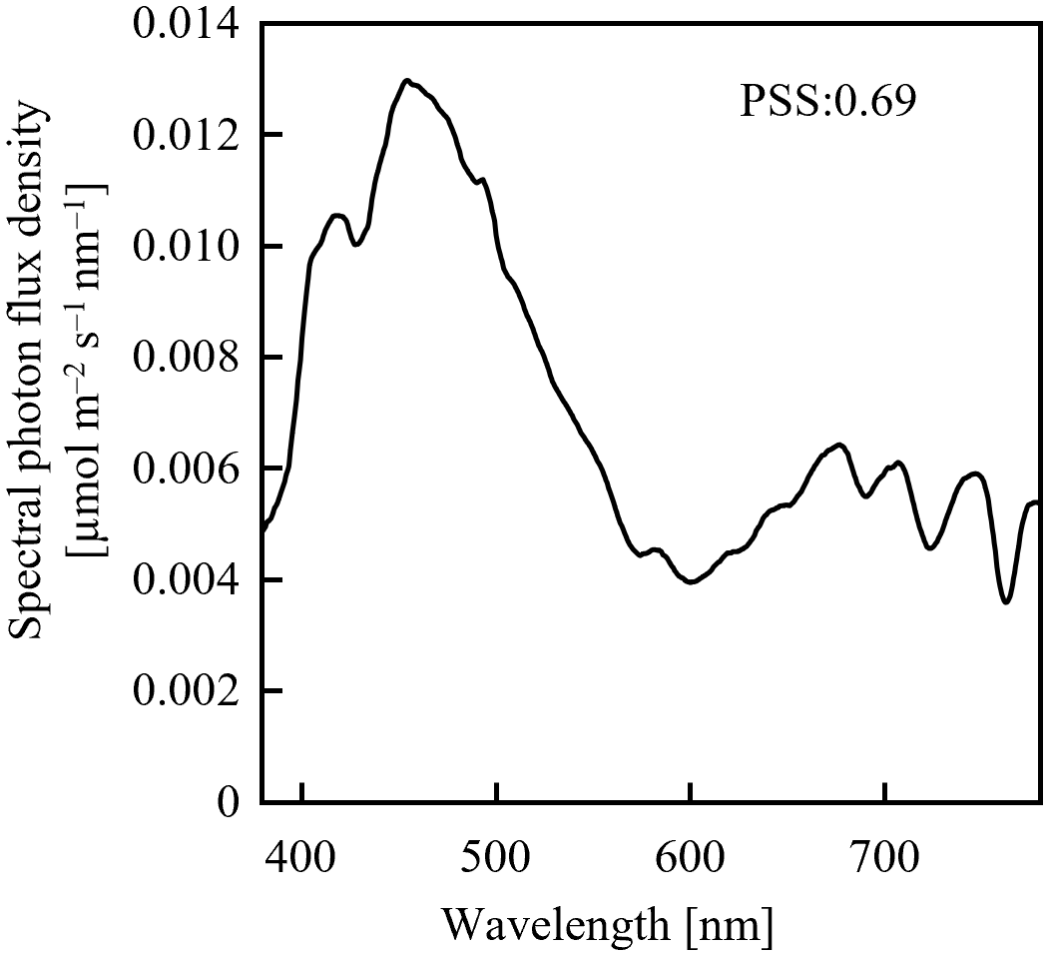
Spectral photon flux density distribution of sunlight measured on a clear June day in Chiba, Japan at 18:50 (sunset at 18:50).

Assuming an initial PSS of 0.69, its temporal change was estimated, as shown in **Figure 1**, after irradiation with far-red LED light having a PFD of 10 µmol m^-2^ s^-1^ or 20 µmol m^-2^ s^-1^. 0.69 was the PSS under a sunlight spectrum (**Figure 2**) in Chiba, Japan (35.8°N, 140.0°E) measured with a photometric sensor (LA-105; Nippon Medical & Chemical Instruments Co., Ltd) at 18:50 on a clear day in June 2020 (sunset at 18:50). A far-red LED (IR749JQ-5AJ2-F1; Toricon, Shimane, Japan), with a 745-nm peak wavelength and a 32-nm full-width at half-maximum (FWHM) was used for the spectral data. The rate of PSS change was estimated to be twice as fast for the far-red LED light irradiation having a PFD of 20 µmol m^-2^ s^-1^, relative to that with 10 µmol m^-2^-s^-1^ (**Figure 1**). Because the rate constant for the PSS change was assumed to be proportional to the PFD (Equation 5), it was estimated that equal integrated light doses with the same RSPD resulted in equal PSS changes. Under the above conditions, 90% of the PSS change was calculated to be complete at a 10 µmol m^-2^ s^-1^ × 345 s = 3.45 mmol m^-2^ EODFR dose and 99% complete at a 6.90 mmol m^-2^ EODFR dose.

If the sunlight was continued after the EODFR completion, the effect on PSS regulation was small because the PSS was expected to increase after EODFR, according to Equation 5. Sunlight at Chiba, including red light, was still present after sunset with a PPFD greater than 2 mmol m^-2^ (data not shown), and its effect on the phytochrome could not be ignored. Therefore, to reduce the PSS, the EODFR should be started after sunset, when sunlight PFD is sufficiently small.

### 3.2 Monochromatic LED light irradiation

**Figure 3:**
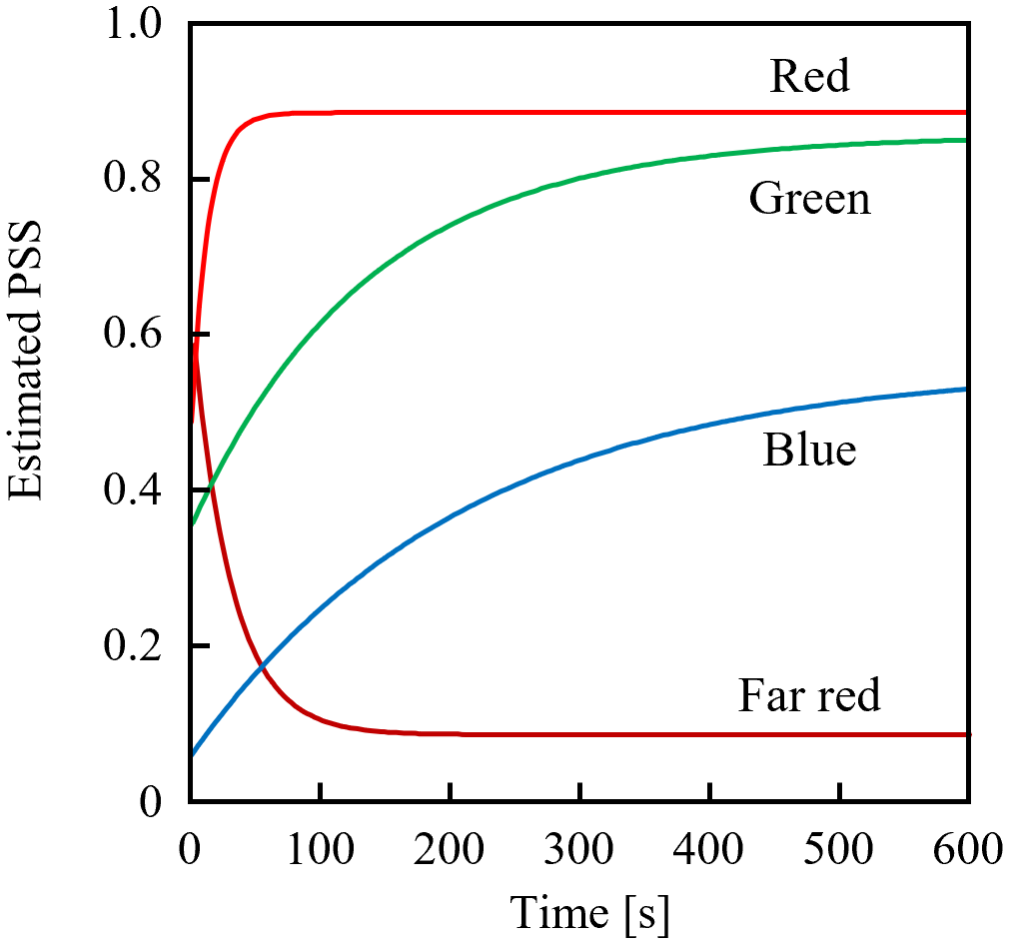
Estimated temporal changes in the phytochrome steady state (PSS) from initial PSS values 0.5 away from the equilibrium value when exposed to blue, green, red, or far-red light-emitting diodes.


**Figure 3** shows the estimated temporal changes in the PSS under various monochromatic LED sources with PFDs of 10 µmol m^-2^ s^-1^. The blue LED (OSUB5161P; Optosupply Limited, Hong Kong) had a 471-nm peak wavelength and a 25-nm FWHM; the green LED (OSPG5161P; Optosupply Limited, Hong Kong) had a 531-nm peak wavelength and a 36-nm FWHM; the red LED (OS5RKA5B61P) had a 632-nm peak wavelength and a 20-nm FWHM; and the far-red LED was as described above. The initial PSS value was 0.5 different from the steady-state value. The rate constants of PSS changes were approximately 2:3:33:14 when irradiated with blue, green, red, and far-red light, respectively. The estimated rate of PSS change was the product of the difference between the current and steady-state values and the rate constant, and was not affected by whether the PSS increased or decreased. If the P_fr_ dark reversion was ignored (detailed in Sec. 3.3 below), the time required to complete x% of the PSS change could be expressed in Equation 11 and was only affected by the value of Equation 12.

(11) $$\begin{array}{c} t=-\frac{\log\left(1-\frac{x}{100}\right)}{a+b} \end{array}$$

(12) $$a+b=\sum_{\lambda =300}^{800}E_{\text{λ}}({\text{σ}}_{\text{rλ}}+{\text{σ}}_{\text{frλ}})\;$$

**Figure 4:**
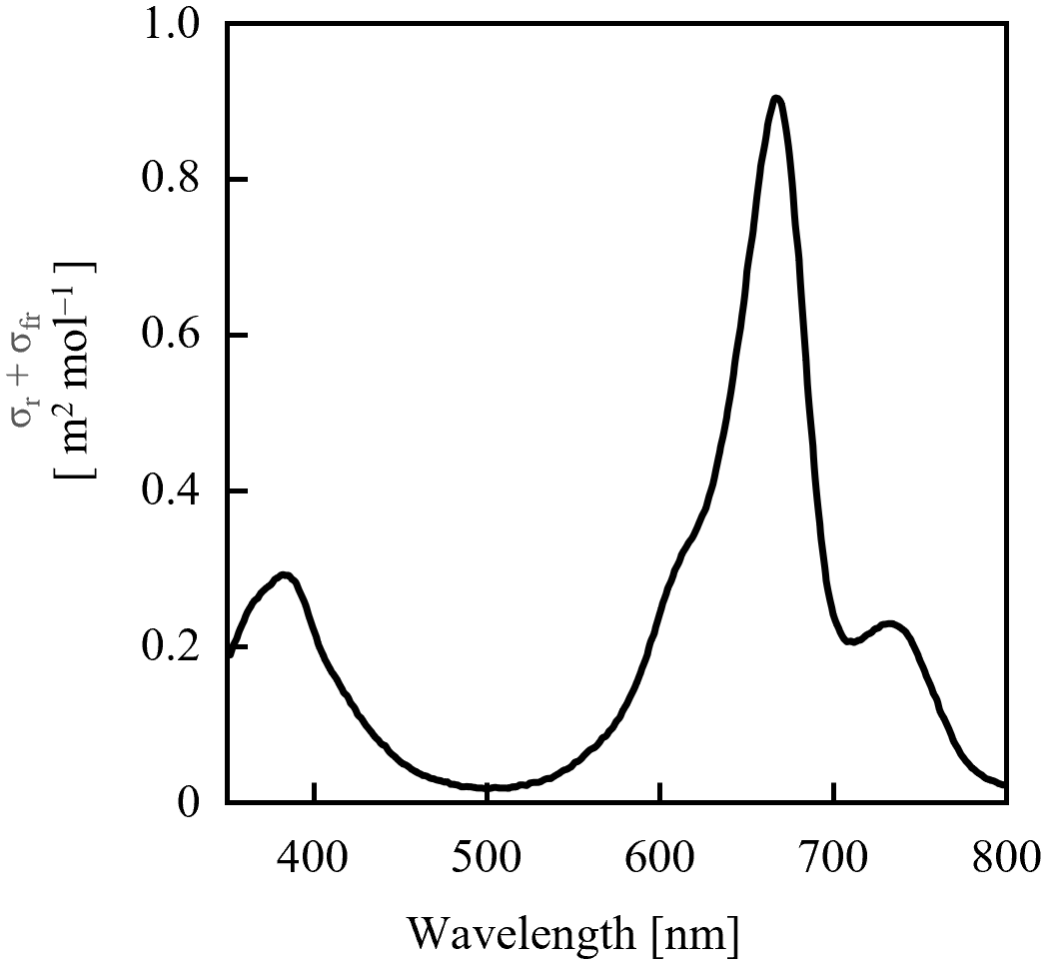
Sum of phytochrome photochemical cross-sections of P_r_ (σ_rλ_) and P_fr_ (σ_frλ_) calculated from the data of Sager et al. (1988).

Because the values of σ_rλ_ + σ_frλ_ were comparable in the 700–740 nm range (**Figure 4**), it was estimated that the dose of far-red LED light required for sufficient PSS changes did not differ significantly depending on the selected LED. Conversely, for red light, the rate of PSS change was estimated to be approximately half that at 600 nm and 700 nm, relative to red light containing more intensity at 660 nm.

### 3.3 Dark reversion

The conversion of P_fr_ to P_r_ in the dark is referred to as P_fr_ dark or thermal reversion (see review of Klose et al., 2020). Kasperbauer et al. (1964) estimated the rate of P_fr_ dark reversion to be 0.8% min^-1^ from the flowering response in Chenopodium rubrum L. This value was comparable to that reported by Klose et al. (2015) for a 60-min half-life of the PSS. These values were comparable to the effect of 0.3-µmol m^-2^ s^-1^ far-red LED light irradiation.

## 4 Discussion

It was calculated that 90–99% of the PSS change was completed with a 3.45–6.90-mmol m^-2^ EODFR dose, although the values were slightly affected by the RSPFD of the light. This was comparable to the results of Chia and Kubota (2010), who reported that EODFR effects on tomato morphology were almost saturated at 2–4 mmol m^-2^ doses, and the results of Yang et al. (2012) who reported that the EODFR effect on hypocotyl elongation of pedunculate squash was saturated at 4 mmol m^-2^.

Zou et al. (2021) reported that the EODFR effects on the leaf areas and dry weights of lettuce saturated at approximately 10 mmol m^-2^; but those effects slightly increased with increased EODFR doses up to 180 mmol m^-2^. Based on the present model, the PSS slightly approached a steady-state value as the FR dose increased. However, the calculated difference in the PSS after 10-mmol m^-2^ and 180-mmol m^-2^ EODFR was less than 0.1%, and it was unlikely that this small difference had any effect. These results could be attributed to overlapping leaves. Zou et al. (2021) conducted cultivation for about 29 days after sowing, which is a longer period than other reports. Thus, the canopy should have grown great, and the lower leaves should have been exposed to lower-PFD light penetrating the upper leaves. More EODFR doses outside the canopy may have been needed to saturate the response of the lower leaves. It is difficult to estimate the phytochrome response of the entire canopy. The PSS in each leaf of the canopy would be estimated by using the light-environment distribution model of the canopy, in addition to the present PSS estimation model. This may lead to advances in future environmental light-control techniques that take into account differences in SPFD attributed to locations within the canopy.

Changes in the total phytochrome amount were not considered in the model here. The important physical quantity as a signal to plants was not the PSS but the absolute amount of active P_fr_ (Schmict and Mohr, 1982). Schäfer and Mohr (1974) estimated that under greater far-red light intensity, the total amount of phytochrome was reduced. Consideration of changes in the total phytochrome amount may lead to more precise estimates of phytochrome-mediated responses. Also, the PSS here was calculated using the phytochrome data of Sager et al. (1988) derived from oats (Mancinelli, 1986). Because light is also absorbed by plant pigments other than phytochromes, the model equation could be improved, especially with regard to the estimation of absolute PSS values.

When attempting to control the phytochrome reaction in plants via artificial lighting, the plant response is not always linear with the PSS value. The response may occur when the PSS exceeds a threshold value and may be saturated at a certain PSS. Furthermore, plants are affected by other photoreceptors, and those reactions may interact with phytochrome reactions. Hence, artificial lighting in horticulture should be designed with those considerations as well as the cost of light sources.

## 5 Conclusions

Based on previously reported data on isolated phytochromes, a model equation was developed to estimate temporal change in the PSS with respect to initial PSS values and the SPFD of the light. The calculated estimations were consistent with previous studies that examined dose requirements of end-of-day far-red light irradiation. The model also enabled estimates of the time required for progress in PSS changes up to x %. This model could be used to control plant responses via phytochrome reactions induced by artificial lighting.

## Data availability statement

The original contributions presented in the study are included in the article/**Supplementary Material**. Further inquiries can be directed to the corresponding author.

## Author contributions

TJ: Conceptualization, Data curation, Methodology, Writing – original draft.

## Supplementary material

Supplementary Table 1 A calculator that outputs the estimated time course in PSS.
